# The isothiocyanate sulforaphane induces respiratory burst oxidase homologue D‐dependent reactive oxygen species production and regulates expression of stress response genes

**DOI:** 10.1002/pld3.437

**Published:** 2022-09-06

**Authors:** Andrés Arruebarrena Di Palma, Enzo A. Perk, Martín E. Carboni, Carlos García‐Mata, Hikmet Budak, Mahmut Tör, Ana M. Laxalt

**Affiliations:** ^1^ Instituto de Investigaciones Biológicas CONICET ‐ Universidad Nacional de Mar del Plata Mar del Plata Argentina; ^2^ Museo Argentino de Ciencias Naturales “Bernardino Rivadavia”–CONICET Buenos Aires Argentina; ^3^ Montana BioAgriculture, Inc. Missoula Montana USA; ^4^ Department of Biology, School of Science and the Environment University of Worcester Worcester UK

**Keywords:** Arabidopsis, biotic stress, heat shock proteins, NADPH oxidase, oxidative stress, plant defense, RBOHD, reactive oxygen species, RNAseq, sulforaphane

## Abstract

Sulforaphane (SFN) is an isothiocyanate‐type phytomolecule present in crucifers, which is mainly synthesized in response to biotic stress. In animals, SFN incorporated in the diet has anticancer properties among others. The mechanism of action and signaling are well described in animals; however, little is known in plants. The goal in the present study is to elucidate components of the SFN signaling pathway, particularly the production of reactive oxygen species (ROS), and its effect on the transcriptome. Our results showed that in *Arabidopsis*, SFN causes ROS production exclusively through the action of the NADPH oxidase RBOH isoform D that requires calcium as a signaling component for the ROS production. To add to this, we also analyzed the effect of SFN on the transcriptome by RNAseq. We observed the highest expression increase for heat shock proteins (HSP) genes and also for genes associated with the response to oxidative stress. The upregulation of several genes linked to the biotic stress response confirms the interplay between SFN and this stress. In addition, SFN increases the levels of transcripts related to the response to abiotic stress, as well as phytohormones. Taken together, these results indicate that SFN induces an oxidative burst leading to signaling events. This oxidative burst may cause the increase of the expression of genes such as heat shock proteins to restore cellular homeostasis and genes that codify possible components of the signaling pathway and putative effectors.

## 1 INTRODUCTION

Plants develop adaptative strategies to survive natural environmental challenges. These strategies could involve physicals (e.g., spines and shells) and/or chemicals (resins, proteins, and secondary metabolites). Secondary metabolites are low‐molecular weight compounds synthesized by plants that are not essential for life but quite important because they are involved in many physiological process and responses (Pusztahelyi et al., 2015; Tiwari & Rana, 2015; Ullah et al., 2017).

The Brassicales order produce an effective chemical defense mechanisms (specific for this class) based on a group of metabolites known as glucosinolates (Fahey et al., 2001). Glucosinolates are found in broccoli, cauliflower, cabbage, and the model plant *Arabidopsis* (Halkier & Gershenzon, 2006) and are very stable water‐soluble molecules, substrates of myrosinases (Fahey et al., 2001; Kliebenstein et al., 2005). Myrosinase and glucosinolates are physically separated in different cell types and when tissue is broken, both components get together and glucosinolates get hydrolyzed (Wittstock & Burow, 2010). Myrosinase‐mediated glucosinolate hydrolysis may lead to the generation of many types of products including nitriles, thiocyanates and isothiocyanates. Among the glucosinolate breakdown products, isothiocyanates possess the highest chemical reactivity (Pastorczyk & Bednarek, 2016). In *Arabidopsis*, around 40 biologically inactive glucosinolates have been identified in several accessions and the most abundant is glucoraphanin that upon myrosinase action produce is the isothiocyanate sulforaphane (SFN) (Ferber et al., 2020, and reviewed in Burow & Halkier, 2017). Glucosinolates are stored at high levels (>130 mM) in specific cells called S‐cells localized in the phloem cap along the vasculature and along the leaf margins (Koroleva et al., 2010).

Isothiocyanates are mainly related to defense in plants, generated upon the attack by insects and/or pathogenic microorganisms (Fan et al., 2011; Malka et al., 2020). In addition, in undamaged tissue the hydrolysis of glucosinolates and the presence of isothiocyanates, possibly with a role in an eventual attack by pathogens has also been reported (Wittstock & Burow, 2010). On the other hand, different pathogens have strategies to inhibit this isothiocyanates‐mediated response. For example, the pathogenic fungus *Sclerotinia sclerotiorum* synthesizes an isothiocyanate‐hydrolase that reduces the levels of isothiocyanates, making the plant more susceptible to the infection (Chen et al., 2020). This evidence suggests an important role for isothiocyanates in the pathogenesis processes, both in defenses of the plant and in infection by the pathogen. They also have functions as allelochemicals, in sulfur homeostasis, in water transport, heat tolerance, stomatal regulation, apoptosis, growth inhibition, and in the signaling of different processes (Bones et al., 2015).

Specifically, SFN production was reported in cells treated with bacterial effectors, simulating biotic stress (Andersson et al., 2015; Fan et al., 2011) and during pathogen infections (Wang et al., 2020). Furthermore, SFN is capable of inhibiting the type III secretion system in the pathogenic bacterium *Pseudomonas syringae*, and plants deficient in SFN synthesis are more susceptible to infection (Wang et al., 2020). Finally, SFN acts not only upon cell rupture after and infection but also when it is applied exogenously generates a maximized response to pathogens in plants (Schillheim et al., 2017). Plants pre‐treated with SFN showed greater resistance to infection by the virulent oomycete *Hyaloperonospora arabidopsidis* (Andersson et al., 2015; Schillheim et al., 2017). In plants the signaling pathway of SFN is not known in detail. It has been reported that SFN causes a decrease in the cellular content of glutathione and as a consequence of increase in the redox potential (Andersson et al., 2015; Ferber et al., 2020). Likewise, SFN produces covalent modifications in histone H3, causing the unpacking of chromatin and the activation of the expression of the defense genes WRKY6 and PDF1.2 (Schillheim et al., 2017). It has not yet been determined whether SFN is related to the production of reactive oxygen species (ROS) in leaves, a common response to attack by pathogens. In particular, Hossain et al. (2013) reported that degradation products of glucosinolates like allyl‐isothiocyanate (isothiocyanate group), 3‐butenenitrile (nitriles group), and ethyl thiocyanate (thiocyanates group) generate ROS production in guard cells. Calcium oscillations were generated in response to SFN treatments, although the mechanisms of action of SFN to trigger both responses were not known in detail. Recently, Afrin et al. (2020) shows that SFN induces stomatal closure as a consequence of ROS production, GSH depletion, and calcium concentration transient spikes.

The ROS generation is usually a response to the recognition of infection and the sign of the activation of plant defenses against biotic stress (Torres, 2010). ROS have several signaling functions, which mediate the establishment of multiple responses by regulating the expression of numerous genes. Several enzymes including plasma membrane NADPH oxidases (NADPHox) and cell wall peroxidases may be responsible for the generation of ROS detected in the apoplast. Generally, NADPHox are activated by calcium influx through plasma membrane Ca^2+^ channels (Torres, 2010). Within this group of proteins, respiratory burst oxidase homologue D (RBOHD) is responsible for a rapid production of ROS upon the recognition of pathogens (Kadota et al., 2015). RBOHD is activated by Ca^+2^ binding, by independent and Ca^+2^‐dependent kinases, and by phosphatidic acid (Kadota et al., 2015; Zhang et al., 2009). Here we report that exogenous SFN application induced ROS production via the RBOHD in leaf discs in a calcium‐dependent manner. In addition, a large increase in the expression of heat shock protein (HSP) genes is detected, and also, genes associated with response to oxidative stresses are modified.

## 2 RESULTS

### 2.1 *Sulforaphane induces ROS production in* Arabidopsis

**FIGURE 1:**
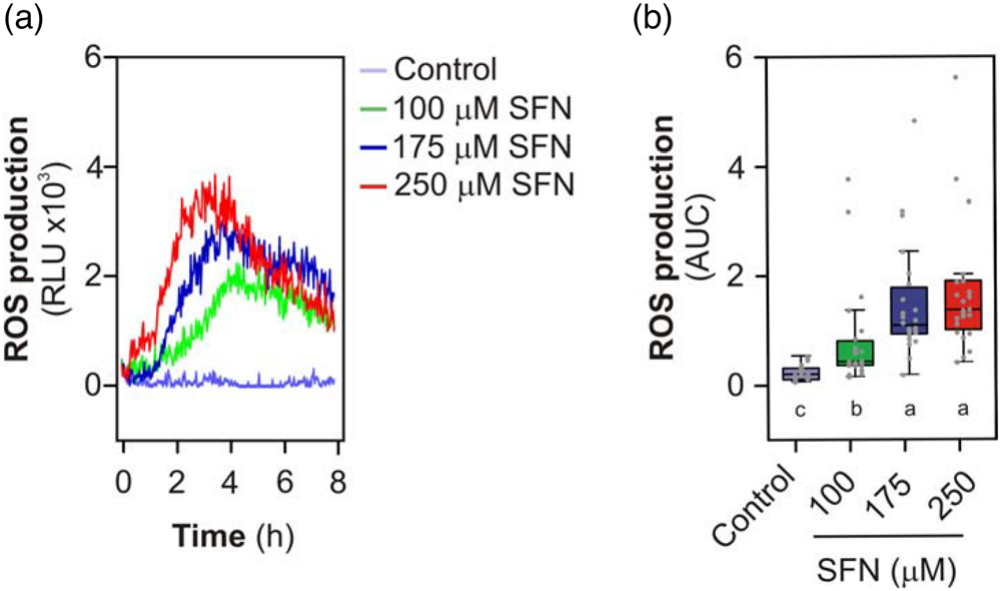
Sulforaphane induces reactive oxygen species (ROS) production in *Arabidopsis* leaf disc. (a) The production of ROS was measured with peroxidase/luminol based method. *Arabidopsis* leaf disc were treated with sulforaphane (SFN) or deionized water (Control) for 8 h, taking light emitted by leaf disc every 2 min with integration time of 1 s. Graph shows representative curves of relative light units (RLU) from one independent experiment. (b) Total peroxide production was calculated integrating the areas under the curves (AUC) obtained from each leaf disc (*n* = 8). Gray dots correspond to data from each individual leaf disc from three independent experiments. Data were compared statistically by non‐parametric Kruskal–Wallis test with Dunn post hoc test (*p* < .05). Different letters indicate statistical differences.

In order to know whether SFN induces plant‐defense responses, we tested if SFN induces ROS production in *Arabidopsis* leaf discs. Leaf disc of 4 weeks old Arabidopsis plants were treated with 100, 175, or 250 μM of SFN and ROS production was quantified by peroxidase/luminol based method according to Bisceglia et al. (2015). A time course measurement of ROS production shows a peak at 3 h for 250 μM of SFN (Figure 1a). A shift in the time of the peak is observed for lower SFN doses. The dose response effect of SFN was quantified as the area under the curve, showing a significant increase from 100 to 175 μM (Figure 1b). Lower SFN concentration led to weak or non‐reproducible ROS production (data not shown).

### 2.2 Sulforaphane induces cytosolic calcium increase that is required for ROS production

**FIGURE 2:**
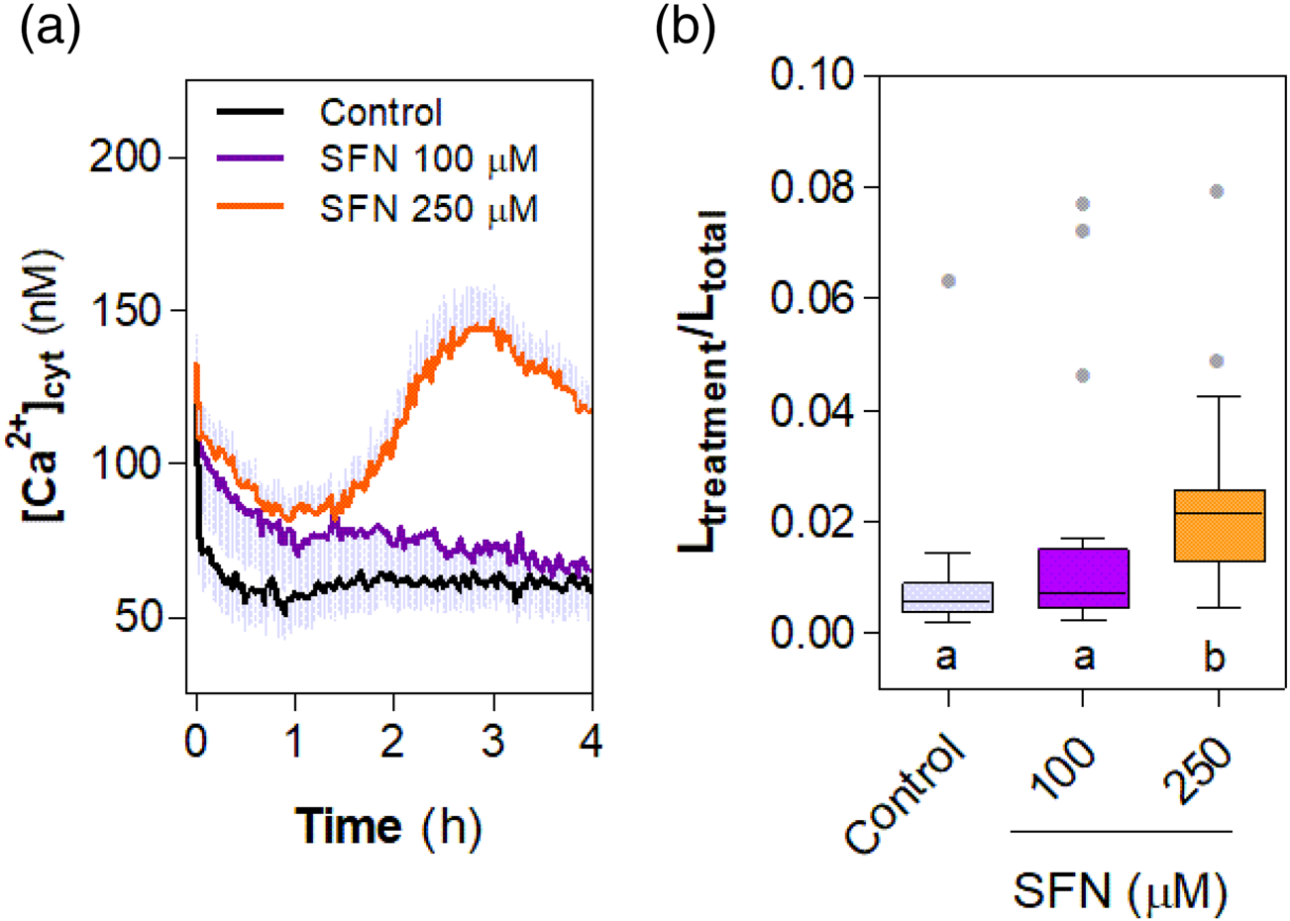
Sulforaphane (SFN) increases cytosolic calcium concentrations. Cytosolic calcium concentrations were monitored using aequorin expressing Col‐0 seedlings preincubated with coelenterazine and treated with 100 or 250 μM of SFN or deionized water as control. Light emitted by seedlings (*n* = 12) was measured every 2 min with integration time of 1 s for 4 h. (a) Cytosolic calcium oscillation upon SFN treatment. The figure shows a representative assay. Luminescence was transformed to calcium concentration using Tanaka et al. (2013) equation. (b) Normalized light emitted by *Arabidopsis* seedlings along 4 h of measurement of two independent experiments (L_treatment_ represent accumulated light generated by treatment and L_total_ the light emitted by seedling after trigger discharge using exogenous calcium). Data were compared statistically by non‐parametric Kruskal–Wallis test with Dunn post hoc test (*p* < .05). Different letters indicate statistical differences.

An increase in cytosolic calcium concentration is generally involved in regulation of ROS production upon pathogen attack (Vadassery & Oelmüller, 2009). Since SFN induces ROS production (Figure 1), we then analyzed if cytosolic calcium increases upon SFN treatments. Therefore, we used *Arabidopsis* plants expressing aequorin (AEQ) protein to monitor cytosolic fluctuations in calcium concentration. As observed in Figure 2, 250‐μM SFN induces an increase in cytosolic calcium concentration after 3‐h treatment.

**FIGURE 3:**
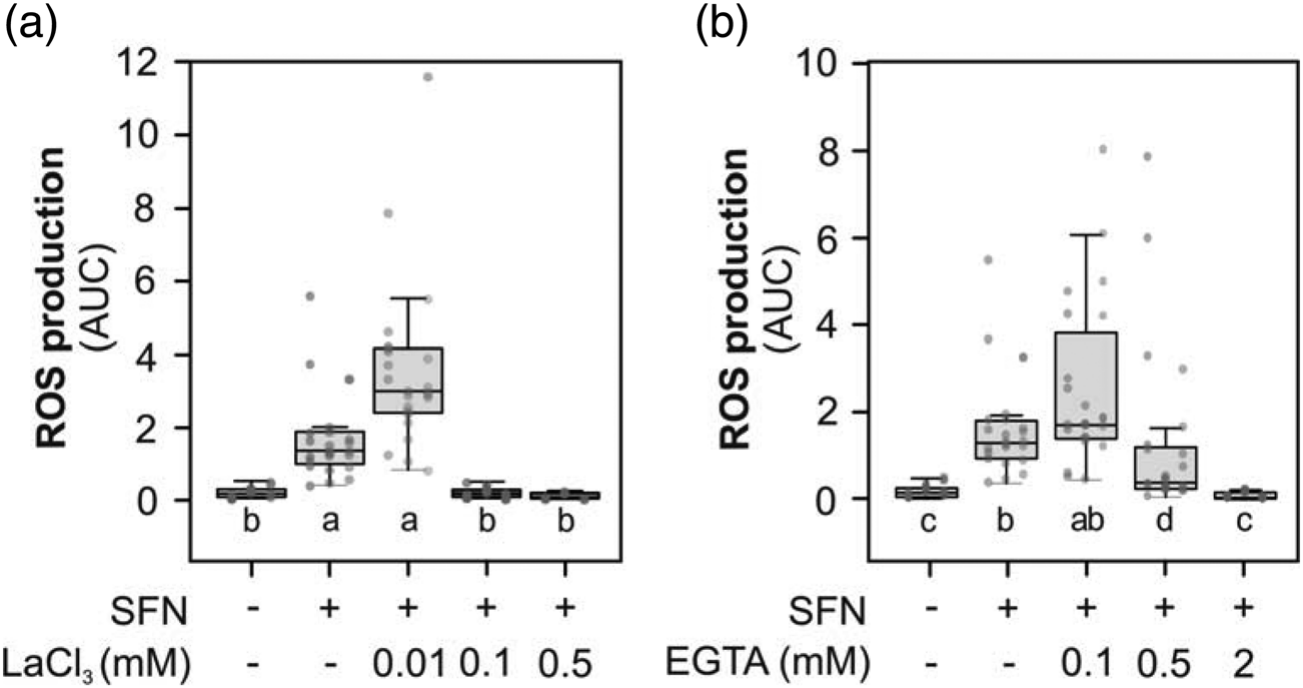
Calcium is required for reactive oxygen species (ROS) production trigger by SFN. *Arabidopsis* leaf discs were treated with sulforaphane (250 μM, SFN indicated with +) or deionized water (−) during 8 h in presence of increased concentration of LaCl_3_ (a) or EGTA (b). Production of ROS was measured with peroxidase/luminol based method. Light emitted by leaf disc was taken every 2 min with integration time of 1 s. Total ROS production was calculated integrating the areas under the curves obtained from each leaf disc (area under the curve, AUC). Gray dots correspond to data from each individual leaf disc from three independent experiments. Data were compared statistically by non‐parametric Kruskal–Wallis test with Dunn post hoc test (*p* < .05). Different letters indicate statistical differences.

In line with the increase in cytosolic calcium concentrations induced by SFN (Figure 2), we then studied the effect of calcium channel blocker LaCl_3_ (Figure 3a) or calcium chelating agent EGTA (Figure 3b) on peroxide production triggered by SFN. On both cases, .1 or .5 mM of LaCl_3_ or .5 or 2 mM of EGTA drastically reduced SFN induced ROS production (Figure 3). The results suggest the Ca^2+^ influx is required for ROS production induced by SFN.

### 2.3 RBOHD *is involved in ROS generation by SFN treatment*

**FIGURE 4:**
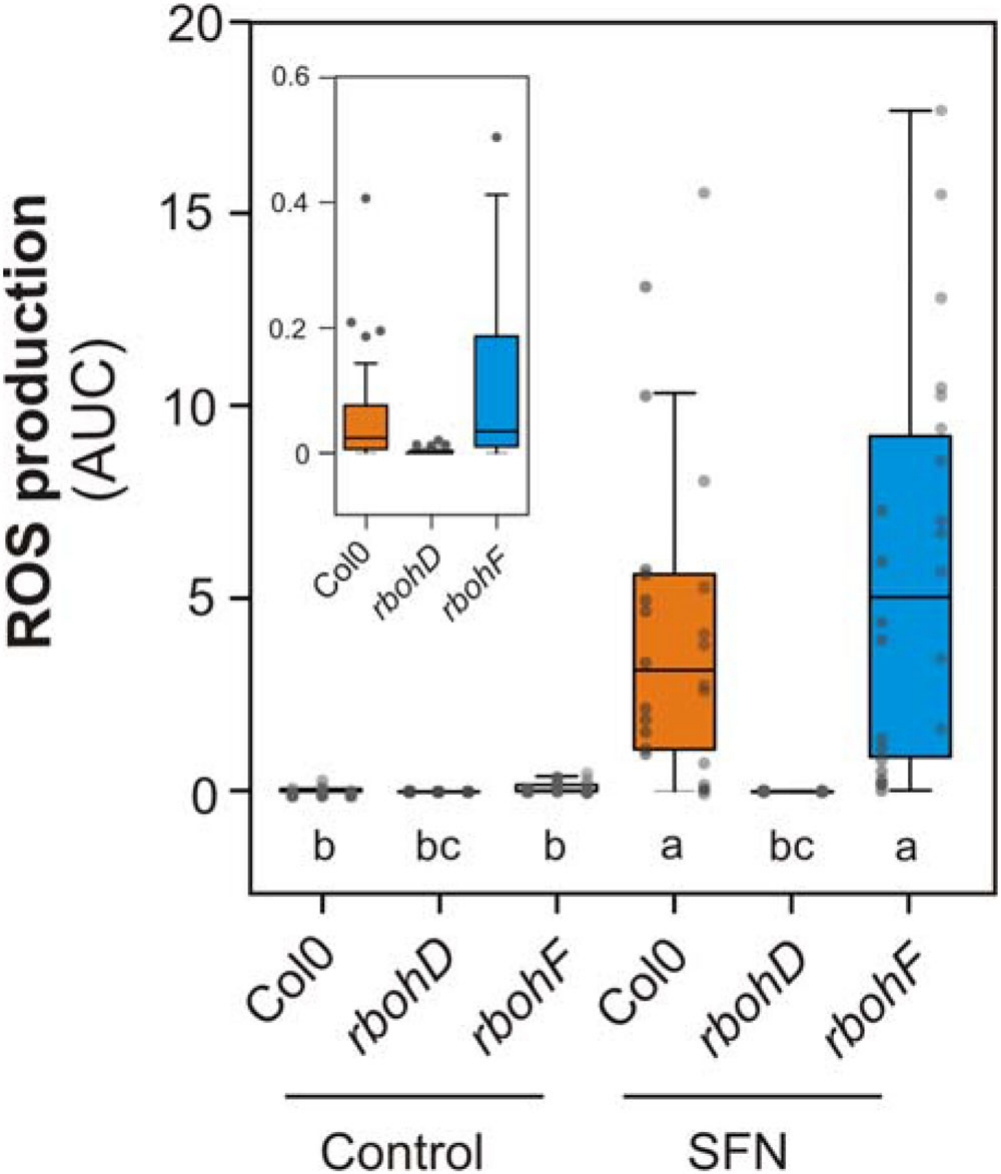
Respiratory burst oxidase homologue D (RBOHD) is involved in reactive oxygen species (ROS) production triggered by sulforaphane. ROS production was measured with peroxidase/luminol based method. *Arabidopsis* leaf disc (*n* = 12) of Col0 WT, *rbohD* or *rbohF* mutants treated with sulforaphane (250 μM, SFN) or deionized water (control) for 8 h. Light emitted by leaf discs was taken every 2 min with integration time of 1 s. Total ROS production was calculated integrating the areas under the curves (AUC) obtained from each leaf disc. Gray dots correspond to data from each individual leaf disc from two or three independent experiments. Data were compared statistically by non‐parametric Kruskal–Wallis test with Dunn post hoc test (*p* < .05). Different letters indicate statistical differences. Inset shows in appropriate scale data from controls.

The role of SFN as a defense molecule, its involvement in ROS production, and the requirement of calcium for this response led us to evaluate if NADPH oxidases were involved in ROS production. In *Arabidopsis*, respiratory burst oxidase homologue D (RBOHD) is responsible for a rapid production of ROS against the recognition of pathogens (Kadota et al., 2015). We then studied SFN‐induced ROS production in the *rbohD* and *rbohF* mutants (Figure 4). Figure 4 shows that RBOH isoform D is required for SFN‐induced ROS production. The *rbohF* mutant was also evaluated, showing the same SFN‐induced ROS production as the wt, indicating that this isoenzyme is not involved in this response.

### 2.4 Changes in gene expression induced by SFN

To evaluate the effect of SFN on Arabidopsis at the transcriptomic level, we performed an RNA‐seq analysis of *Arabidopsis* seedlings treated with two SFN doses. We found 94 differentially expressed genes (DEGs) in 400‐μM SFN treatment being 70 of them upregulated and 24 downregulated. For 800‐μM SFN we found 189 DEGs, 110 upregulated and 79 downregulated (Tables S1 and S2).

**FIGURE 5:**
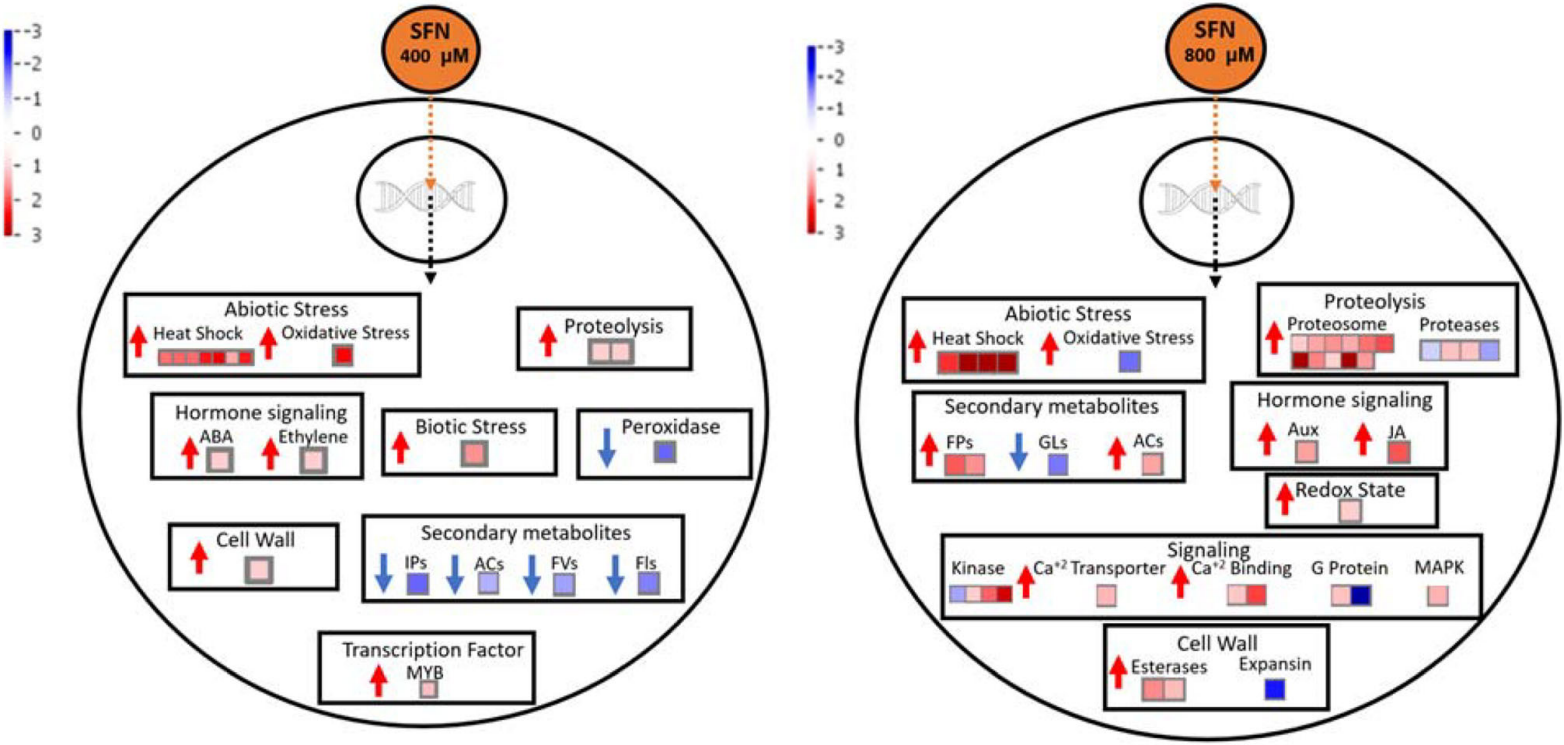
Differentially expressed genes (DEGs) classified in functional groups. DEGs from 400‐ and 800‐μM sulforaphane (SFN) were analyzed by MapMaN. In red, DEGs with increased expression level and in blue with decrease expression level. FPs: Phenylpropanoids, GLs: Glucosinolates, ACs: Anthocyanins, Aux: Auxins, JA: Jasnomic acid, IPs: Isoprenoids, FVs: Flavones, FLs: Flavonoids. The unit of the displayed scale is Log_2_FC. Accession numbers are indicated in Table S3.

To have a global idea of the SFN's effect in both treatments we performed an analysis using MapMan Software that groups the different DEGs by their principal function. The analysis showed that mainly the DEGs were grouped in stress related processes (Figure 5 and Table S3). We focused on functional groups of DEGs related to stress because the previous evidence (Afrin et al., 2020; Andersson et al., 2015; Fan et al., 2011; Hossain et al., 2013; Schillheim et al., 2017; Wang et al., 2020) links SFN actions with plant‐pathogen interaction and ROS production.

In 400‐μM SFN treatments, we found eight upregulated DEGs in the group of abiotic stress, seven are *HSP* (*AT3G46230/AT3G12580/AT5G51440/AT2G29500/AT1G53540/AT5G12030*) and the other one is *MSRB9* (*AT4G21850*), which is linked to oxidative stress response with possible function to stabilize oxidized proteins. Two DEGs are related to proteolysis (*AT1G23980* and *AT1G78100*). One DEG related to cell wall strengthening (*AT2G01850*) and one MYB factor which enhances the expression of stress related genes (*AT2G21650*). Two upregulated DEGs were related to ABA (*AT1G69700*) and ethylene (*AT5G07580*) signaling, which may link SFN effect with other phytohormones. A really interesting result was that all the DEGs related to secondary metabolites were downregulated, all of them generally related to plant defense. Another downregulated DEG is *PER70* (*AT5G64110*), a peroxidase with unknown function.

On the other hand, similar to the transcripts induced in 400‐μM SFN treatment, in 800‐μM SFN, there were DEGs related to abiotic stress, more specifically related to heat shock response and oxidative stress. However, it is interesting to notice that only six DEGs were shared between the two SFN doses used; nevertheless, they are related to the same functional groups (BINs). There was a high upregulation of DEGs related to protein degradation, most of them in the proteasome pathway. Also, there were many DEGs related to the cell wall, two esterases (*AT1G67830* and *AT3G14310*) and one expansin (*AT3G55500*), indicating it could result in cell wall strengthening. Interestingly, there were many DEGs related to signaling, like kinases, calcium transporters, calmodulins, and redox sensors, in correlation to our findings of ROS production and increase in cytosolic Ca^2+^ concentrations upon SFN treatments.

We then analyzed the top 10 upregulated and downregulated DEGs for both treatments. Results are shown in Table 1. In regard to the response to 400‐μM SFN, 4 out of the 10 highly overexpressed genes correspond to *HSP*. Another gene related with re‐folding of denatured protein methionine‐sulfoxide dismutase (*MSBR9*) was overexpressed as well. The other induced gene was *ZIFL1*, involved in the auxin entry pathway regulating the abundance of PIN2 auxin transporter (Remy et al., 2013). Several of them have unknown function (position 5 to 8). The most repressed DEGs encode proteins with various biological functions, including growth, weakening of the cell wall, photosynthetic performance, and production of secondary metabolites. Contrary to what was observed for the 10 most overexpressed DEGs, a relationship, at least obvious, between the different SFN repressed DEGs was not found.

**TABLE 1:** Top 10 upregulated and downregulated DEGs for (a) 400‐ or (b) 800 μM SFN treatment

400 μM			
Upregulated			
ID	Gene name	Log_2_FC	Function
AT1G53540	HSP 17.6C	2.56	Molecular chaperone
AT4G21850	MSRB9	2.41	Oxidative stress response
AT2G29500	HSP 17.6B	2.31	Molecular chaperone
AT5G52640	HSP 90.1	2.29	Molecular chaperone
AT5G13750	ZIFL1	2.27	Transporter
AT1G52825		2.20	Sporulation specific protein (putative)
AT5G55900		2.04	Sucrase/ferredoxin (putative)
AT5G49800		1.99	Contains a lipid binding domain
AT1G23890		1.93	Contains NHL domain
AT3G12580	HSP70	1.85	Molecular chaperone

400 μM			
Downregulated			
ID	Gene name	Log_2_FC	Function
AT1G70980	SYNC3	−2.14	Class II aminoacyl‐tRNA and biotin synthetases superfamily protein
AT4G24540	AGL24	−2.12	Encodes a MADS‐box protein involved in flowering.
AT3G14550	GGPS3	−1.87	Encodes a protein with geranylgeranyl pyrophosphate synthase activity involved in isoprenoid biosynthesis.
AT5G64110		−1.86	Peroxidase superfamily protein
AT3G55080		−1.83	SET domain‐containing protein
AT4G30140	CDEF1	−1.74	Cutinase.
AT5G07130	LAC13	−1.69	Laccase. (putative)
AT5G58770	CPT4	−1.61	AtCPT7 synthesizes medium‐chain polyprenols of approximately 55 carbons in length.
AT4G22490		−1.60	Bifunctional inhibitor/lipid‐transfer protein/seed storage 2S albumin superfamily
AT4G27370	VIIIB	−1.57	Member of myosin‐like proteins

800 μM			
Upregulated			
ID	Gene name	Log_2_FC	Function
AT1G77000	SKP2B	7.21	F‐box protein
AT5G10650	JUL1	6.80	RING‐type E3 ubiquitin ligase
AT5G08710	RUG1	6.01	Regulator of chromosome condensation (RCC1) family protein
AT3G12580	HSP70	5.89	Molecular chaperone
AT5G52640	HSP90.1	5.69	Molecular chaperone
AT5G18970	AWPM‐19 LIKE	5.35	AWPM‐19‐like family protein
AT5G12390	FIS1B	5.16	Organelle division
AT5G06750	APD8	4.80	Protein phosphatase 2C family protein
AT3G28210	PMZ	4.80	Zinc finger protein (putative)
AT2G29525	IPCS3	4.76	Inositol phosphorylceramide synthase

800 μM			
Downregulated			
ID	Gene name	Log_2_FC	Function
AT1G55040	SED1	−6.27	Unknown
AT3G46668		−5.96	Unknown
AT2G40316		−5.95	Autophagy‐like protein
AT4G35810		−5.07	2‐oxoglutarate (2OG) and Fe (II)‐dependent oxygenase superfamily protein
AT4G30140		−3.81	Cutinase
AT2G33980	NUDT22	−3.31	Nudix hydrolase homologue 22
AT1G18075	MIR159B	−3.22	Encodes a microRNA that targets several MYB family members.
AT3G01850		−2.86	Aldolase‐type TIM barrel family protein
AT1G28710		−2.80	Nucleotide‐diphospho‐sugar transferase family protein
AT4G13720		−2.75	Inosine triphosphate pyrophosphatase family protein

*Note*: Ten days old *Arabidosis* seedlings sprayed with 400‐ or 800‐μM SFN treated for 30 h. Selection of the first 10 induced and repressed DEGs. The table shows the IDs, the name taken from the TAIR database (https://www.arabidopsis.org/) and the Log_2_FC with respect to control.

The first 10 DEGs overexpressed by the 800‐μM SFN treatment show a great diversity of functions. Although *HSPs* appear, they do only twice, compared to the situation observed for 400‐μM SFN. The most overexpressed DEG (*SKP2B*) has a role on the cell cycle, degrading a negative regulator of cyclins (KRP1) (Ren et al., 2008). Another interesting DEG that correlates with ROS production is *FISSION‐1B*, involved in the peroxisome cleavage process. This process may be associated with modifications in the cellular environment (oxidative environment) generated by various types of biotic and abiotic stresses (Pan & Hu, 2011). Finally, DEGs such as *APD8* (phosphatase), *PMZ* (redox‐dependent zinc finger protein), and *IPCS3* (inositol phosphorylceramide synthase) reveal the activation of the sensing and signaling mechanism, which could be participating in the response pathway to SFN.

## 3 DISCUSSION

Sulforaphane belongs to a diverse family of plant defense compounds, the most abundant one being isothiocyanate in *Arabidopsis* (Fahey et al., 2001). However, it is not known exactly the molecular mechanisms of how SFN exerts its effect in plants. Few evidences showed that SFN alter the plant cellular glutathione content leading to a redox imbalance, although this effect is dependent on the concentration used and the application method (Andersson et al., 2015; Ferber et al., 2020). Previous experiments performed in our lab on tomato cell suspensions indicated that SFN induces ROS production (Figure S1). Here, we first focus on the role of SFN in the production of ROS. The generation of ROS is a key step in the response of plants to pathogens (Torres, 2010). SFN induces ROS production via RBOH isoform D (Figures 1 and 4). RBOHD is a key component in signaling against biotic and abiotic stresses, participating in the control of cell death, lignification induced by cell wall damage (Kadota et al., 2015; Miller et al., 2009). In general, ROS production via RBOHD triggered by pathogen effectors is fast, between seconds to minutes (Kadota et al., 2015). As a classical example, ROS production upon flg22 recognition occurs within 5–10 min (Kadota et al., 2015). In the case of ROS production trigger by SFN, the action of RBOHD is slow compared to flg22 response. ROS peak production occurs at 2–3 h after treatment. This late response triggered by SFN could be related to the internalization kinetics of the molecule. Interestingly, Ferber et al. (2020) shows that it takes about 2–3 h for 100‐μM SFN to completely ingress into plant cells. This timing of entry of SFN to cells correlate to maximum ROS production for same SFN concentration in our experimental conditions. Late ROS production driven by RBOHD was triggered too by another electrophile molecule, the nitrolipid nitro‐oleic acid (NO_2_‐OA, Arruebarrena Di Palma et al., 2020). However, ROS response triggered by NO_2_‐OA shows a by‐phasic kinetics of production and differs from SFN. Recently Schellenberger et al. (2021) found a similar late ROS production driven by RBOHD upon bacterial rhamnolipids (not electrophile molecules) treatment in Arabidopsis. The specific role of this long and late ROS production in plant physiology needs to be investigated more deeply.

RBOHD activation is mainly mediated by Ca^+2^ and phosphorylation by protein kinases, both Ca^+2^‐dependent and independent (Kadota et al., 2015; Kimura et al., 2020; Ogasawara et al., 2008). Using aequorin reporter *Arabidopsis* lines, we showed that SFN induce an increase in cytosolic calcium concentration (Figure 2). The timing of cytosolic calcium increase occurs within hours (2–3 h), a clear different timing observed upon pathogen effector recognition. In addition, preliminary evidence generated in our lab using tomato cell suspensions shows that ROS production is diminished by using both extracellular calcium chelators and protein kinases inhibitors (Figure S1). In line with this, calcium influx from the extracellular is required for ROS production in response to SFN, since using an extracellular calcium chelator (EGTA) or a channel blocker (LaCl_3_) impairs ROS signal induced at 250‐μM SFN (Figure 3) as well as for 100 μM SFN (Figure S2).

Another analysis that we perform was an RNA‐seq to evaluate SFN effects at transcriptomic level. In both SFN treatments, induction of genes associated with HSP was observed (Figure 5 and Table 1). In line with our results, Ferber and colleagues (Ferber et al., 2020) found an increased response of genes associated with HSP by treatment with SFN and this response is dependent on the action of the transcription factor *HSFA1*, one of the DEGs that was also detected in our RNA‐seq analysis (Table S2). Whether HSFA1 is a SFN‐target remains unknown. Independent results showed that aliphatic allylisothiocyanate molecule induces high expression of HSPs (Kissen et al., 2016). Due to the electrophilic nature, SFN could alter different still unknown cellular targets. Nevertheless, the induction of genes associated with HSP could be related indirectly via the ROS production (Haq et al., 2019). Faced with an oxidative outbreak, among other possible consequences, proteins can undergo modifications of their residues leading to conformational changes and possible alteration of their functions. These misfolded proteins are the target of action of HSPs, described mainly as molecular chaperones, leading these defective polypeptides to the degradation pathway or to their correct refolding (Divya et al., 2019). In relation to this, in 800‐μM SFN treatments numerous DEGs linked to protein ubiquitination, proteasome and protease activity, as well as peroxisome dynamics were up‐regulated, showing that cell recycling processes could be triggered by SFN. Others interesting DEGs found were *DJ1A* and *MRSB9*, which main function is related to HSPs, refolding unfolded proteins. Cell wall strengthening is a common response to ROS production (Torres, 2010). In our analysis, DEGs related to repressed lignin degradation and cutinase were found, while genes related to lignin synthesis and cell wall pectin dimethyl esterification were overexpressed (Figure 5). Both aspects are of great interest to continue with the investigation of the effects of SFN on cell wall.

At the top 10 upregulated DEGs in 400‐μM SFN treatments, an interesting DEG is *ZIFL1* related to auxin entrance to cell (Remy et al., 2013). ZILF1 regulate PIN2 abundance on cell membrane (Remy et al., 2013). Kissen et al. (2016) found that isothiocyanates reduce the expression of several auxin induced genes. On the other hand, Urbancsok et al. (2017) using auxin‐reporter lines in *Arabidopsis* found no regulation by isothiocyanate treatments. In addition, Katz, Nisani, Sela, et al. (2015) and Katz, Nisani, Yadav, et al. (2015) studying indole‐3‐carbinol (a molecule derived from hydrolysis of isothiocyanate indol‐3‐methyl isothiocyanate) show less PIN1 and miss localization of PIN2 transporters. Relationship between SFN with auxin signaling as well as its relation with another phytohormones like ABA, JA, and ethylene (with representative DEGs found in both treatments, Figure 5) could suggest a relation between these actors. With respect to signaling events altered by SFN treatment, RNA‐seq data show the presence of numerous DEGs related to transport, response, and signaling by calcium, protein kinases, and MAPK (Figure 5), adding further evidence on the involvement of ROS and calcium as second messengers in SFN signaling pathways.

The mechanisms of action of SFN remain an open question. Different mechanisms by which SFN could act are discussed. First, as mentioned above, SFN is an electrophilic molecule. Electrophiles show affinity toward regions rich in electrons, generating a chemical reaction accepting a pair of electrons in order to form a bond (adduct) with a nucleophile (Schopfer et al., 2011). Amino acids that function as nucleophiles are cysteine (Cys) and Histidine (His). In particular, proteins with exposed Cys and cellular glutathione (GSH) could be targets of action of SFN (Schopfer et al., 2011). In this way, Andersson et al. (2015) show that in plants treated with SFN, the concentration of GSH is considerably reduced, although no reference is made to its precise mode of action. On the other hand, Ferber et al. (2020) show that SFN increased SFN‐GSH adduct but without reduction of cellular GSH pool. In animal cell lines, SFN rapidly ingresses to cell, form an adduct with GSH and later, most SFN shifts to form adducts with proteins (Mi et al., 2007, 2011; Nakamura et al., 2018). As occur with other electrophiles (such as nitrolipids) the formation of adducts with specific cellular proteins could alter its function and trigger a response. Regarding ROS production, SFN could be altering the function of various proteins involved in the signaling pathway with functions associated with RBOHD activity. Since Ca^+2^ is required for the activation of this enzyme, SFN could activate pathway by forming an adduct with Cys residues of Ca^2+^ transport channels or even activate proteins that regulate them. Finally, RBOHD itself could be directly activated by SFN. Recently, it was described that persulfidation of Cys825 and Cys890 by H_2_S activates the RBOHD (Shen et al., 2020). If the SFN interacts with any of these Cys in RBOHD, it could modify its activity. Even more, SFN could lead to modification of transcriptome by generating posttranslational modification of some transcriptional factors.

Altogether, our results provide evidence that SFN induce a late ROS production driven specifically by RBOHD that required calcium. In addition, these events, with possible direct electrophilic actions of SFN on cellular targets, could lead directly or indirectly to altered gene expression. This work generates a contribution to the knowledge of the effects of SFN on the physiology of plants. The specific target or targets and the underlaying mechanism of SFN action in plant cells remains an open question.

### 3.1 Experimental procedures

#### 3.1.1 Chemicals and preparation

Pure sulforaphane (LKT Labs, Saint Paul, Minnesota) was solubilized in DMSO (EMSURE, ACS, K46505352 520) to 118‐mM stock concentration. Various concentrations of SFN were prepared in deionized water for treatment. Previously, we check that equivalent dilutions of DMSO in deionized water as a control for each SFN concentration did not induce response, similar to deionized water alone, so for further experiments we use only deionized water as control (with exception of RNAseq assay). Luminol (Sigma‐Aldrich St Louis, USA) and HRP (Sigma‐Aldrich St Louis, USA) were prepared in deionized water. Coelenterazine (Biosynth Carbosynth) were prepared in 98% ethanol. LaCl_3_ (Sigma‐Aldrich St Louis, USA) and EGTA (Sigma‐Aldrich St Louis, USA) were prepared in deionized water and diluted to treatment concentration in deionized water. MS medium was purchased from Sigma‐Aldrich St Louis, USA.

### 3.2 Measurement of ROS in leaf discs of *Arabidopsis* *thaliana*

Seeds from wild‐type *Arabidopsis* Col‐0, *AtrbohD* (mutant knock‐out for AtROHD) and *AtrbohF* (mutant knock‐out for AtROHF) (Torres et al., 2002) were planted in soil (soil:vermiculite:perlite, 3:1:1) and kept at 4°C for 2 days for stratification. Then, they were transferred to growth chamber at 25°C using 8 h light/16 h dark photoperiod for 30 days. Leaf discs of 5.5 mm in diameter were used. The production of H_2_O_2_ was measured with the method based on the reaction of luminol with H_2_O_2_ catalyzed by the peroxidase enzyme according to Bisceglia et al. (2015). *Arabidopsis* leaf discs were treated with SFN or deionized water (control) for 8 h in 98‐well ELISA plates. The light emitted by the leaf disk during this time was recorded as relative light units (RLU) every 2 min, with an integration time of the light emitted of 1 s (as Arruebarrena Di Palma et al., 2020). The total H_2_O_2_ production was calculated integrating the areas under the curves obtained from each leaf disc. For each treatment, between 8 and 12 discs from different plants were used, and the experiment was repeated three times. In the case to analyze the effects of calcium channel blocker (LaCl_3_) and calcium chelator (EGTA), discs were treated with both chemicals as indicated concentration in Figure 3 from the beginning of the measurement. Statistical comparison was made with the Kruskal–Wallis test.

### 3.3 Measurement of cytosolic calcium in *A. thaliana* seedlings

Cytosolic calcium concentrations were monitored using aequorin expressing Col0 seedlings according to Tanaka et al. (2013) protocol. Seedlings were grown on Petri dishes containing MS medium for 10 days and transferred to ELISA plate to perform assay. Seedlings were preincubated with coelenterazine (12.5 μM) and treated with 100 or 250 μM of SFN or deionized water as control. Light emitted by seedlings (*n* = 12) was measured every 1 min with integration time of 1 s for 4 h using a luminometer (Thermo Scientific Luminoskan Ascent Microplate). Luminescence was transformed to calcium concentration using Tanaka et al. (2013) equation. For integration of total light emitted by seedlings were used to normalize light along 4 h of measurement. Data were compared statistically by non‐parametric Kruskal–Wallis test.

### 3.4 Processing of biological material for analysis by RNA‐seq

Ten‐days old seedlings Col‐0 seedlings grown in MS medium were sprayed with SFN in two different concentrations: 400 and 800 μM. DMSO (.8% v/v) was used as a control. For each treatment, three biological replicates were carried out. The pool of complete seedlings (aerial part and root) was processed and RNA was extracted. The samples were sent to the RNAseq service with the ILLUMINA system (Exeter University Sequencing Services, UK).

### 3.5 RNA‐seq data processing

For data processing, as a first step we preprocessed the RNA‐seq raw reads using Trimmomatic116 (parameters ILLUMINACLIP:TruSeq3‐PE‐2.fa:2:30:10 LEADING:20 TRAILING:20 SLIDINGWINDOW:4:20 MINLEN:30 HEADCROP:5) to remove adaptor sequences and low‐quality reads. The clean reads were then mapped to the reference genome using HISAT2 with default parameters. The expression abundance values were calculated using StringTie117, and we averaged the abundance values from the three biological replicates of each sample to obtain levels of gene expression. This protocol was developed in Pertea et al. (2016) and is called new Tuxedo.

After this, the analysis began in the R environment (R Core Team, 2019) where the treatments were compared to find differences in the abundance of the different transcripts. A CSV file was created with the “identification (Replica)” and with the “Treatment.” Then in R the different libraries “RSkittleBrewer” “ballgown” “genefilter” “dplyr” “devtools” “library” “gplots” “ggplot2” were loaded and the file with the quantifications was loaded with the Ballgown function.

A common problem with RNA‐seq data is that genes often have very few or zero counts. A common step is to filter out these types of genes. For this reason, a variance filter was used in which all transcripts with a variance between samples of less than one was eliminated following the indications of the new Tuxedo protocol (Pertea et al., 2016).

The Ballgown stattest function was used to find transcripts that are expressed differently between the groups, that is, the differentially expressed genes (DEG). And in this way the change of each transcript with respect to the control situation (Fold Change, FC) and the *p* value was obtained. We selected as DEGs all the transcripts that had a log_2_FC more than 1 or less than −1.

### 3.6 Analysis in MapMaN

The MapMan program (http://mapman.gabipd.org/web/guest/mapman‐ version‐3.6.0. Thimm et al., 2004) classifies the IDs entered according to their function, in different categories called BIN. Based on these, classifications were possible to determine in which processes are DEGs involved. In this case, this program uses TAIR 9 as the reference genome. Within the program, the function that allows filtering the DEGs according to those involved in stress pathways was used.

## CONFLICT OF INTEREST

The authors have no conflict of interest to declare.

## SIGNIFICANCE STATEMENT

Sulforaphane is a phytomolecule present in crucifers, mainly synthesized in response to biotic stress. The mechanism of action and its putative role as a signal molecule are investigated in this study. Sulforaphane activates Ca^2+^ entry from the extracellular, ROS production via RBOH isoform D activation and expression of heat shock proteins (HSP) and genes associated with the response to oxidative stress.

## Supporting information


**Supplemental Figure 1.** *Sulforaphane (SFN)‐ROS production requires calcium entrance form the extracellular and protein kinase activity in tomato cell suspension.* Tomato cell suspension were treated with 100 μM of SFN. At 3 hs of SFN treatment cells were supplemented with: calcium channel blocker LaCl_3_ (10 mM), extracellular calcium chelator EGTA (10 mM) or protein kinase inhibitor staurosporine (2 μM) and incubated for another hour. ROS production was evaluated by incubating for one hour with 4 μM H_2_DCF‐DA probe. Fluorescence was measured using a fluoroskan over 60 minutes taking data every 2 minutes. Data represents media and standard error of 6 samples of one representative assay.

**Supplemental Figure 2.** Calcium is required for ROS production trigger by 100 μM SFN. Arabidopsis leaf discs were treated with sulforaphane (100 M, indicated with SFN) or water (Control) during 8 hours in presence of .5 mM LaCl_3_ or 2 mM EGTA. Production of ROS was measured with peroxidase/luminol based method. Light emitted by leaf disc was taken every 2 minutes with integration time of one second. Total ROS production was calculated integrating the areas under the curves obtained from each leaf disc (area under the curve, AUC). Samples correspond to data from each individual leaf disc from 2 independent experiments. Data were compared statistically by non‐parametric Kruskal‐Wallis test with Dunn post hoc test (p < .05). Different letters indicate statistical differences.

**Supplemental Table 1:** Differentially expressed genes (DEGs) in 400 μM SFN treatments

**Supplemental Table 2:** Differentially expressed genes (DEGs) in 800 μM SFN treatment.

**Supplemental Table 3:** Accession numbers from DEGs from 400 μM and 800 μM SFN analyzed by MapMaN.

## Data Availability

The RNAseq data SRA accession no: **PRJNA809715.**
