# Supplementary figures and images for: The isothiocyanate sulforaphane induces respiratory burst oxidase homologue D‐dependent reactive oxygen species production and regulates expression of stress response genes
Source: Plant Direct. 2022 Sep 6;6(9):e437. doi: 10.1002/pld3.437 (PMC9448665; doi:10.1002/pld3.437)

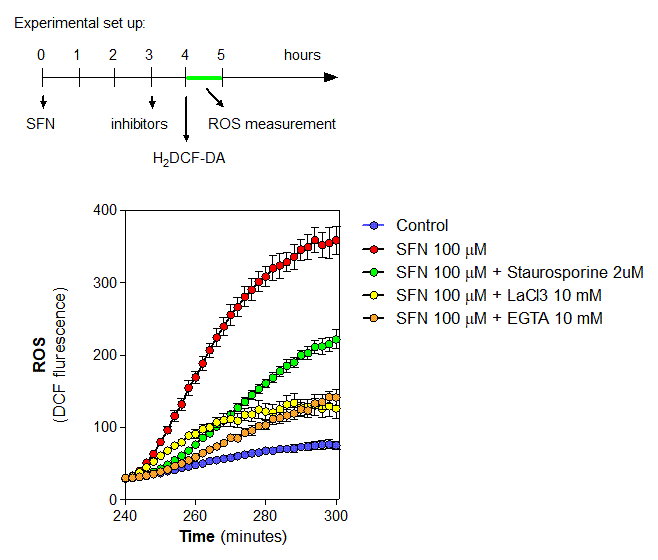

Supplement: Supplementary file 1 — Supplemental Figure 1. Sulforaphane (SFN)‐ROS production requires calcium entrance form the extracellular and protein kinase activity in tomato cell suspension. Tomato cell suspension were treated with 100 μM of SFN. At 3 hs of SFN treatment cells were supplemented with: calcium channel blocker LaCl3 (10 mM), extracellular calcium chelator EGTA (10 mM) or protein kinase inhibitor staurosporine (2 μM) and incubated for another hour. ROS production was evaluated by incubating for one hour with 4 μM H2DCF‐DA probe. Fluorescence was measured using a fluoroskan over 60 minutes taking data every 2 minutes. Data represents media and standard error of 6 samples of one representative assay. [file PLD3-6-e437-s004.png]

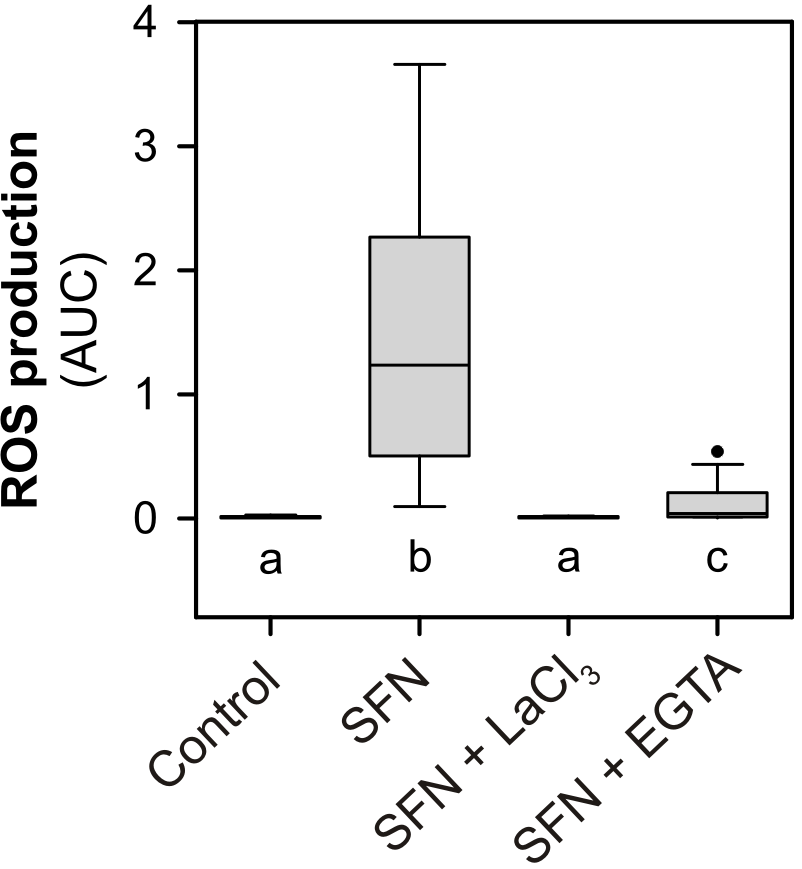

Supplement: Supplementary file 2 — Supplemental Figure 2. Calcium is required for ROS production trigger by 100 μM SFN. Arabidopsis leaf discs were treated with sulforaphane (100 M, indicated with SFN) or water (Control) during 8 hours in presence of .5 mM LaCl3 or 2 mM EGTA. Production of ROS was measured with peroxidase/luminol based method. Light emitted by leaf disc was taken every 2 minutes with integration time of one second. Total ROS production was calculated integrating the areas under the curves obtained from each leaf disc (area under the curve, AUC). Samples correspond to data from each individual leaf disc from 2 independent experiments. Data were compared statistically by non‐parametric Kruskal‐Wallis test with Dunn post hoc test (p < .05). Different letters indicate statistical differences. [file PLD3-6-e437-s003.png]
